# A descriptive qualitative study of adolescent girls’ well-being in Northern Finland

**DOI:** 10.3402/ijch.v73.24792

**Published:** 2014-10-01

**Authors:** Varpu Wiens, Helvi Kyngäs, Tarja Pölkki

**Affiliations:** Institute of Health Sciences, Oulu University, Oulu, Finland

**Keywords:** well-being, girl, gender, descriptive, content analysis

## Abstract

**Background:**

Previous studies have shown that girls present welfare-related symptoms differently than boys and that the severity of their symptoms increases with age. Girls living in Northern Finland experience reduced well-being in some aspects of their lives. However, the opinions of girls on these matters have not previously been studied.

**Objective:**

The aim of this study was to describe girls’ well-being in Northern Finland.

**Method:**

This is a descriptive qualitative study. The participants were 117 girls aged between 13 and 16 who were living in the province of Lapland in Finland and attending primary school. Data were collected electronically; the girls were asked to respond to a set of open-ended questions using a computer during a school day. The responses were evaluated by using inductive content analysis.

**Results:**

Four main categories of girls’ well-being were identified: health as a resource, a beneficial lifestyle, positive experience of life course, and favourable social relationships. Health as a resource was about feeling healthy and the ability to enjoy life. A beneficial lifestyle was about healthy habits and meaningful hobbies. Positive experience of life course is related to high self-esteem and feeling good, safe, and optimistic. Favourable social relationships meant having good relationships with family and friends.

**Conclusions:**

To the participating girls, well-being was a positive experience and feeling which was revealed when they interact between their relationships, living conditions, lifestyle, and environment. Knowledge about girls’ description of their well-being can be used to understand how the girls themselves and their environment influence their well-being and what can be done to promote it.

Finnish Lapland is located in the North Calotte and Barents region. It extends over a large area and its settlements are often separated by long distances. In addition, it is home to a variety of communities, identities, cultures and ways of living. The well-being of individuals living in Northern Finland depends on their living conditions and levels of social exclusion (1). Girls living in Finnish Lapland have more health-related problems in certain areas than their peers in other parts of the country (2), and the rates of substance abuse among young girls in Northern Finland have increased in recent years (3).

The definition of well-being has different interpretations and is thus unclear, and a range of similar terms such as happiness and life satisfaction are used interchangeably (4). Dodge et al. (5) regard well-being as a balance point between an individual's resource pool and the challenges she/he faces. Their definition is based on the concept of a set point of well-being, the inevitability of equilibrium/homeostasis and a fluctuating relationship between challenges and resources.

Various studies have demonstrated that there are sex-related differences in health and well-being (6,7): girls experience more and varied symptoms than boys, and seem to exhibit more symptoms as they age (8–15).The finding that girls are exhibiting more adverse symptoms cannot be regarded as a transient phenomenon (16). Because knowledge about adolescents’ own definitions of well-being is partly narrow (17) and adolescents conceptualize well-being differently (18,19), consultations with young people could provide valuable information that would enable the design of effective interventions (18–20). This has also been emphasized by UNICEF's Convention on the Rights of the Child: children have the right to have their say in matters that concern them (21). Young people are sensitive to how they are involved and heard in matters that concern them (22).

We therefore set out to answer the following research question: how do girls living in Northern Finland describe their own well-being? It was anticipated that our findings would shed new light on adolescents’ well-being and be useful in guiding the design of programmes for its reinforcement.

## Material and methods

### Participants

A descriptive qualitative approach was adopted in this study because the aim was to investigate descriptions of participants’ own well-being. Girls aged between 13 and 16 who lived in the province of Lapland, Finland, and attended primary school were recruited to the study without regard for ethnic background. Purposive sampling (23) was used to ensure that the sample included girls with diverse backgrounds and perspectives in order to attain rich and varied writings. In the province of Lapland, there are 39 primary schools to which a research permit was sent. From these schools, 17 granted a research permit and from them 9 schools that were willing to take part eventually participated. Between them, the 9 participating schools had approximately 720 female pupils aged between 13 and 16. Of these female pupils, 118 responded to our assignment, and 1 answer was rejected because it did not address the research question. In total, 117 responses suitable for analysis were obtained.

The mean age of the participants was 14.2. Most of them lived in detached houses in rural areas of Lapland with their families. Nineteen percent of the girls had divorced parents and 3 of the girls lived in foster homes.

### Ethical considerations

Before the study was begun, it was approved by Northern Ostrobothnia Hospital District Ethics Committee. Because the participating girls were aged between 13 and 16, written consent was obtained from the girls themselves, their parents and the education boards of their communities, as required by Finland's Medical Research Act. During the research, attention was paid to human dignity, which includes participants’ consent, voluntariness and maintaining anonymity. The participants’ anonymity was ensured by assigning ID numbers to their submissions that could not be linked back to their identities. In addition, the submissions were not made available to anyone other than the researchers (23).

### Data collection

Writings were chosen as a data collection method, because it was thought to be a good way to reach girls in a vast and sparsely populated province (23). One objective of collecting writings was trying to get a description from the reality of life and experience from the girl's point of view. A pilot study involving 28 girls was conducted in the spring of 2012, before the start of the main data collection exercise. The written assignment was then refined and modified based on the responses obtained during this pilot study. Detailed instructions regarding the conduct of the study were sent out to the participating schools’ principals and teachers in advance. Data were collected through responses to open-ended question (23,24) that were gathered by electronic means from the schools around the province of Lapland in Northern Finland in 2012. The written assignment to the girls was as follows: describe what well-being is to you. In addition, the girls were asked to write information about their circumstances and background (how old are you, what kind of family do you have, what is your school like and what is your home and your home area like).

The participating girls completed the written assignments under their teachers’ supervision during school hours and then submitted the completed assignments to the researchers via the Webropol online survey software system using their school's computers. Writings were collected in the springtime (n=62) and fall (n=55) of 2012.

### Analysis

**Fig. 1:**
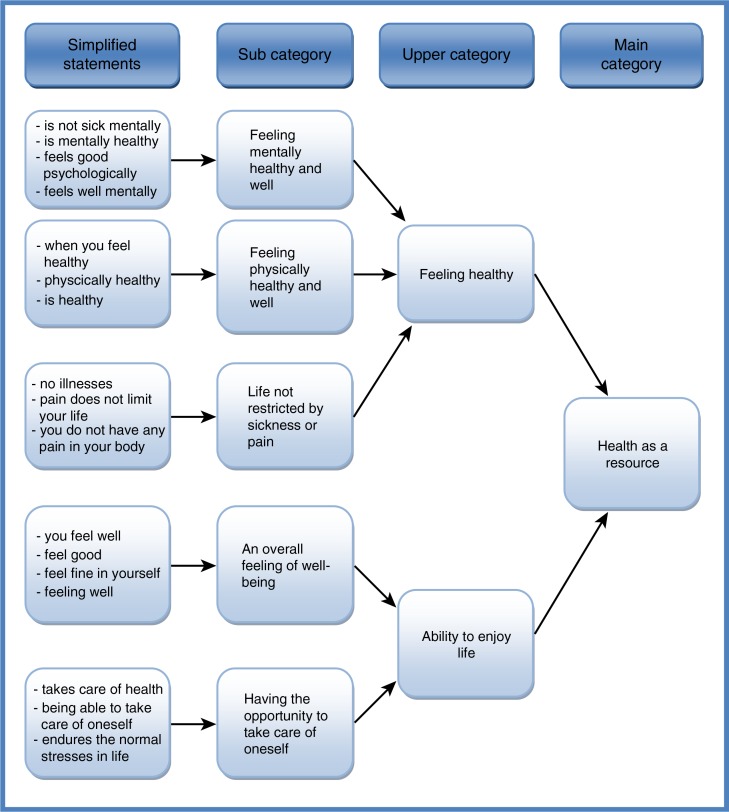
Illustration of the data analysis process showing the grouping of subcategories to form the main category “Health as a resource.”

The data were analysed using inductive content analysis (25). The units of analysis were words or statements that related to the same central meaning. In the first stage of the analysis, each girl's writings were read from beginning to end several times to ensure that the analyst had a clear grasp of their overall contents. The contents were then reduced by converting the girl's original expressions into simplified expressions. Expressions with similar meanings or which dealt with related topics were grouped into subcategories, each of which was assigned a descriptive name. Subcategories with similar meanings were then grouped to form upper and main categories, which were named using content-characteristic words (25). This process of abstraction was continued until new categories could be no longer formed (Fig. 1).

## Results

Four main categories of well-being were identified within the girls’ writings: health as a resource, a beneficial lifestyle, positive experience of life course, and favourable social relationships.

### Health as a resource

**Fig. 2:**
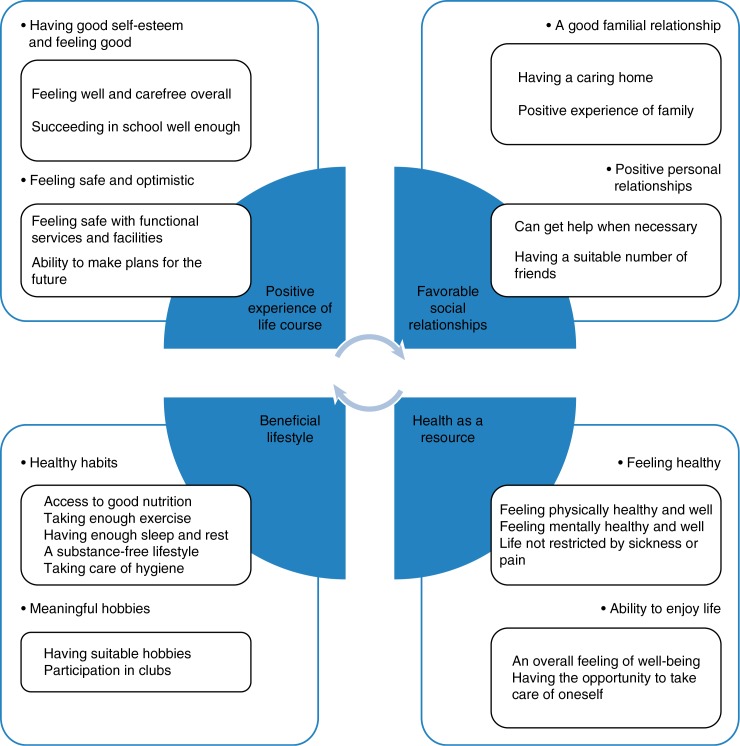
Description of girls’ well-being in Northern Finland.

The girls regarded health as a resource that enables them to live good lives. This category had 2 subcategories: feeling healthy and the ability to enjoy life (Fig. 2). These included meaning units relating to physical and mental health and well-being.

The *feeling healthy* contained meaning units referring to being in good physical health without any restrictions imposed by sickness or pain. The girls considered good physical health to encompass both physical health and the feeling of being physically healthy.

… you are healthy and there are no chronic diseases. – T8

Well-being is physical and not just mental. Physical well-being is important, and it means a lot to me. If I am sick or in pain, I'm not at my best. – T34

Good mental health was also regarded an important component of overall well-being. The girls’ writings suggested that they define good mental health as a comfortable state of mind that enables psychological well-being with no mental illness.

Also mental well-being is part of well-being, to be outgoing and not whining and having good spirits. – T33

*The ability to enjoy life* was based on a holistic sense of feeling good and being able to take care of oneself. A holistic sense of feeling good refers to feeling well in both body and mind in a way that makes one able to cope well, physically and mentally. The opportunity to take care of oneself refers to a capacity to endure the normal stresses and strains of life, and to be contented.

In my opinion, well-being is when you are feeling good but it is also about healthy and positive thinking. – T53

… well-being is cheerfulness and happiness. When you are feeling well, you are able to enjoy life. – T111

### Beneficial lifestyle

The beneficial lifestyle category contained 2 sub categories: healthy habits and meaningful hobbies (Fig. 2). These included meaning units relating matters trying to manage physical and mental well-being.

The *healthy habits* contained meaning units relating to appropriate nutrition, participating in essential exercise, having enough rest, leading a substance-free lifestyle (i.e. not smoking or drinking alcohol), and taking care of one's personal hygiene. Appropriate nutrition was understood to meaning having enough healthy food. On the contrary, the girls wanted to enjoy eating tasty food without feeling guilty.

Part of well-being is eating healthy and right. The objective of well-being is to be healthy and have a normal weight. – T110

To do some jogging, to eat healthy food and to indulge without feeling guilty. – T6

The healthy habits also included meaning units relating to having enough sleep and rest. Feeling unstressed and being able to relax were particularly important to the girls.

You have to remember to relax and sleep well every day. – T17

The *meaningful hobbies* contained meaning units relating to each individual girl's hobbies and interests that had positive effects on their feelings and mental state. The girls mentioned various hobbies including a range of sports and different clubs.

Moving around in your leisure time so that you just don't lie around in the house all day long and sit in front of the screen. Being enthusiastic about moving your body and feeling well. – T33

Hobbies like hip-hop, dicso- and showdancing and youth theater. – T23

### Positive experience of life course

The positive experience of life course category had 2 subcategories: having good self-esteem and feeling good, safe and optimistic. These included meaning units relating to a healthy level of self-esteem, a positive outlook on life and feeling safe and optimistic (Fig. 2).

*Having good self-esteem and feeling good* contained meaning units relating to doing well in school, feeling good and carefree as a whole and self-realization.

Feeling good is an overall sense that things are going well. The girls felt that things were going well if they had no worries and were able to make themselves visible to and heard by other people. Feeling good in this context relates to being able to do the things one wishes to and enjoys doing. In general, this subcategory contains meaning units relating to feeling happy, having a high self-esteem, and feeling loved.

That one feels safe and being loved. – T9

When one is satisfied with herself and does not feel necessary to change anything about herself. – T14

The feeling of doing well in school was associated with the achievement of personal goals. It was motivated by a range of factors and was not linked exclusively to numerical grades. In particular, it was associated with the ability to cope with the requirements and expectations of attending school, and the ability to attend school without being bullied.

Can go to school in high spirits and knowing that there is at least a few people who smiles at you and greets you. – T21

When I get that feeling that I'm doing well at school. – T8

… and that there is no bullying in school. – T38

The *feeling safe and optimistic* contained meaning units relating to the girls’ communities and living environments. Active, functional services and facilities made girls feel safe within these environments and generated feelings of safety and optimism. Access to health care, education and social security were all regarded as key components of safe environments. It is also about feeling safe in the place where you live and in the community that you live in.

It's hard to define well-being is difficult, but to me it is when I feel

safe in the environment where I live. – T108

Good health care system, opportunity for education and equality. – T43

Feeling optimistic also encompassed meaning units relating to the ability to make plans for the future: it is partly about having dreams and the anticipation of doing pleasant things such as taking a summer vacation.

Enjoying things you are doing and having dreams. – T99

Is able to do things you want. – T96

### Favourable social relationships

The favourable social relationships category had 2 subcategories: good familial relationship and positive personal relationships. They contained meaning units associated with positive familial and personal relationships (Fig. 2). The girls considered it was important to have good relationships with their families.

A *good familial relationship* was understood as one that provides a positive and caring environment. Families give support, love and encouragement. At its best, home was seen as a safe place where the girls could relax, prosper and pass time. It was also considered important for a good home to provide a place to live and adequate food and clothing.

In my opinion, well-being means that things are all right with your family and the relationships between your family members are OK. – T40

I think that part of well-being is loving family. – T14

That you are doing well with the family. – T38

Good relationships with friends were widely described as being essential for well-being. The *positive personal relationships* contained meaning units relating to having a suitable number of friends and being able to get help and support from them when necessary. There was no specific number of friends that was considered important, but it was important for the girls to have as many friends as they felt they should have, and for their friendships to be trusting. The girls also considered it important to nurture and cultivate their friendships.

When you are having enough friends in your own opinion. – T8

Well-being is when I spend time with my friends. – T17

Girls also considered it important to know that they had access to help when they experienced difficulties in life. Knowing where to go for such help was regarded as a key component of well-being; feeling well was understood as something that requires support and help.

You might need help and support – and you usually do – from outside,

but you have a lot of influence over what help you can get. – T113

If someone is having troubles, she is being helped. – T4

## Discussion

### Discussion of the results

The aim of this research was to describe girls’ well-being in Northern Finland. The participants provided extensive descriptions of the different aspects of well-being according to their own definitions, which ranged from simple survival and the handling of everyday tasks to self-actualization and the ability to realize one's dreams. In this study, we were interested in the girls’ well-being, and not taking into account the determinants of ethnicity, although this study involved girls living partly in the same geographical areas as the Sami people. Therefore, it is interesting that the contributory factors of well-being identified by the girls who participated in this study were similar to those reported by a sample of Indigenous Circumpolar youths (26), particularly with respect to the perceived importance of a close relationship with one's parents, affection and praise, a safe family environment and a sense of being valued. The WHO-5 Well-Being Index measures mental well-being from the perspective of positive quality of life (27), and these 5 items (felt cheerful and good spirits, felt calm and relaxed, felt active and vigorous, woke up feeling fresh and rested and daily life has been filled with things that interest me) in the WHO-5 Questionnaire cover the same themes as to what the girls wrote on what well-being is to them.

The writings showed that well-being was understood holistically and required that one feels both mentally and physically healthy. This seems to be normal for adolescents. Previous studies have suggested that adolescents’ appraisals of their health are shaped by their overall sense of functioning, which includes both physical and non-physical health dimensions (6,28).

Food and eating seemed to have contradictory opinions in relation to well-being. Good food was considered to be important for well-being both in nutritional terms and as an indulgence, but the girls also felt guilty about having bad eating habits. Previous studies have shown that girls’ well-being is affected by dieting and a negative body image, and that girls exhibit greater levels of dissatisfaction with their weight and appearance than boys (14–16,29,30). However, the participants in this study did not express significant dissatisfaction with their bodies; controlling weight and eating was mentioned mainly as a way to stay healthy.

The meaning units within the positive experience of life course category related to well-being in a holistic sense. This may reflect the fact that well-being is multidimensional and depends on several different things. As stated in other studies (31,32), the health of adolescent girls is a complex phenomenon that is extensively interwoven with their broader lives. The girls in this study seemed to appreciate the positive effects of natural environments. Most of the girls described their region of residence positively and in general, the girls felt that they could move around freely within their region. These findings are consistent with those of Wesely and Gaarder (33), who reported that women who participate in outdoor recreational activities reap many physical and emotional benefits from doing so. In this context, in the future it could be beneficial to study the effects of outdoor activities in nature among girls living in Northern Finland. The perception of a safe environment was partly due to the girls’ awareness of the comprehensive services available to them; they seemed content with the services offered by their communities, even though there are in some places long distances to services in Northern Finland.

Doing well in school, being happy with one's body and self-rated good health have all previously been reported to contribute to well-being among girls (34). In our study, schools were mainly mentioned in a positive manner, although the girls did feel pressure and stress from the expectation of being successful in school and getting good grades. The negative comments about school were related to bullying. School does not seem to have a significant impact on how girls perceive their health: no matter what school they attend, their health status seems to be worse than that of comparable boys (15,35).

Our data indicate that friends are extremely important for girls’ well-being, in keeping with previous findings (34). The well-being of girls also requires the support of strong familial relationships, and it may be that the girls took their families’ presence and support for granted. However, it is clear that adolescents’ well-being is closely connected to that of their families, making the family both the source of and solution to many problems in adolescent well-being (36). Because the findings of this study suggest that girls’ well-being is associated with social relationships (friends and family), a supportive and encouraging network would benefit their well-being. In the Finnish health care system, school nurses have traditionally been the ones who take care of school health promotion and disease management. Interestingly, none of the participating girls mentioned their school nurse or school welfare office as sources of help or support.

### Study limitations

The study had some limitations. The first relates to the voluntariness of the data collection process and the integrity of writings. Because the writings were gathered during the school day, it is possible that the girls felt obliged or pressured to answer. Moreover, it is possible that some of them did not respond truthfully when describing their experiences and perceptions. The second limitation relates to the analytical process: some of the writings were ambiguous, and in some cases it would have been reasonable to assign a given meaning unit to multiple categories. The third limitation is that the analysis was not repeated independently by multiple researchers in order to determine the extent of agreement between separate analyses. However, the credibility of the findings was discussed within the authors’ research group (25).

A relatively large sample was needed to achieve saturation of the data. Therefore, purposive sampling was used to ensure that the sampled group consisted of girls with diverse backgrounds (23). A preliminary study involving 28 girls was conducted to evaluate and refine the approach, increasing the trustworthiness of the results obtained in the main analysis. Two rounds of inductive content analysis were performed and both of these yielded similar upper and main categories. To document the trustworthiness of the analytical process, some of the girls’ original sentences are presented in the text (after having been translated from Finnish into English). Because this study is limited to the girls living in Northern Finland, transferability of the results into another context has to be considered carefully.

## Conclusions

Positive experiences and feelings that are generated by interactions between friends and family, good and supportive living conditions and an environment that supports well-being seems to be the essential elements of girls well-being in Northern Finland. It could be said that girls’ well-being is affected by their own behaviour and choices as well as by the social relationships, structural factors, and living conditions in Northern Finland. It would be good to consider whether the unique geographical aspects of this region should be taken into account when promoting girls well-being. And now that we have some knowledge of what well-being is to the girls living in Northern Finland, subsequently it would be interesting to know what factors inhibit or promote their well-being. This could mean more individualized solutions with new innovative ideas for health promotion and disease prevention for girls living in Northern Finland.
